# Incorporating Trauma-Informed and Culturally Competent Care Within Domestic Violence Screening Training for Medical Students

**DOI:** 10.1192/bjo.2024.322

**Published:** 2024-08-01

**Authors:** Iyinoluwa Popoola

**Affiliations:** King's College London, London, United Kingdom

## Abstract

**Aims:**

The link between domestic violence and poor mental health outcomes is well-established, with victims often experiencing anxiety, depression, and post-traumatic stress disorder (PTSD). This study aims to evaluate the current state of Domestic Violence (DV) screening training within the medical curriculum at King's College London, focusing on trauma-informed and culturally competent approaches. The objective is to identify gaps and propose recommendations for a comprehensive and inclusive training program.

**Methods:**

Approved by the King's College London Research Ethics Office, this qualitative study was conducted using an online questionnaire that adopted a 5-point Likert-type scale. The study was conducted among KCL Medical Students (n = 25) to gather opinions on DV screening training, and the responses underwent thematic analysis.

**Results:**

The survey indicated that 92% of participants had not received formal training on DV screening. In addition, 88% lacked guidance on responding to disclosures in a trauma-informed manner and only 8% believed they had training on responding to DV within diverse cultural contexts. The key themes were ‘Addressing Training Gaps and Challenges’, ‘Practical Skills Enhancement’, ‘Cultural Competency and Diversity' and ‘Comprehensive Understanding of Domestic Violence and Abuse’. Students expressed concerns about the lack of clear, direct education on escalating domestic violence cases. Furthermore, some medical students expressed apprehension about inadvertently re-traumatising or offending patients with a different cultural background.

**Conclusion:**

The study highlights deficiencies in the current domestic violence screening training, emphasizing the urgent need for a more comprehensive, trauma-informed, and culturally sensitive curriculum. Recommendations include the incorporation of domestic violence education within the core curriculum and interprofessional education, survivor engagement, and challenging biases through adopting a critical pedagogy approach. These changes aim to enhance the understanding, attitudes, and practical skills of medical students in addressing domestic violence, ultimately contributing to a more inclusive and responsive medical education system.

